# The LazyBox Educational Intervention Trial: Can Longitudinal Practice on a Low-Fidelity Microsurgery Simulator Improve Microsurgical Skills?

**DOI:** 10.7759/cureus.49675

**Published:** 2023-11-29

**Authors:** Michael A Jensen, Archis R Bhandarkar, Megan M. J. Bauman, Cecile Riviere-Cazaux, Kimberly Wang, Lucas P Carlstrom, Christopher S Graffeo, Robert J Spinner

**Affiliations:** 1 Neurosurgery, Mayo Clinic Alix School of Medicine, Rochester, USA; 2 Neurosurgery, The University of Oklahoma Health Sciences Center, Oklahoma City, USA

**Keywords:** randomized educational interventional trial, microsurgery simulator, resident education, low-resource setting, fourth-year medical student education

## Abstract

Introduction

Every surgical trainee must acquire microsurgical skills within a limited timeframe. Therefore, identifying effective educational strategies to help learners attain these skills is crucial.

Objective

Establish the effectiveness of a low-fidelity microsurgery simulator to improve the execution and one’s perception of the difficulty of basic surgical techniques.

Methods

From 2021 to 2022, 24 medical students were randomized to either (1) a treatment group (n=12) that engaged in longitudinal practice on a low-fidelity microsurgery simulator (the LazyBox) or (2) a control group (n=12) that did not practice. Students performed vessel loop ligation, catheter macroanastomosis, and synthetic vessel microanastomosis prior to and six weeks after intervention. Both objective metrics and subjective metrics (Swedish Occupational Fatigue Inventory (SOFI) and Surgery Task Load Index (SURG-TLX)) were obtained.

Results

The treatment and control arms had 1.2 (SD = 2.6) and 2.1 (SD = 2.4) points increase in the vessel loop ligation, respectively (p = 0.39). The treatment and control arms had a 3.4 (SD = 4.1) and 2.9 (SD = 3.6) points increase in the macroanastomosis task, respectively (p = 0.74). In the synthetic vessel microanastomosis task training, the experimental and control arms showed a 5.4 (SD = 8.3) and a 2.9 (SD = 5.6) points increase, respectively (p = 0.30). No differences were found between the groups regarding survey metrics of mental (p = 0.82), temporal (p = 0.23), and physical demands (p = 0.48).

Conclusion

In our randomized educational intervention, we found no significant difference in objective and subjective metrics of microsurgical task performance between learners who did and did not use the LazyBox simulator.

## Introduction

The microsurgical technique is a mainstay amongst numerous surgical subspecialties, including neurosurgery, plastic surgery, and head and neck surgery. Like other surgical techniques, there is a learning curve in developing the specific skills necessary for microsurgery. However, time and resources to practice microsurgical techniques as a resident can be limited, especially given the size and cost of a surgical microscope. These barriers in accessing resources to practice microsurgical techniques are especially poignant for medical students interested in pursuing a surgical subspecialty. These limitations most substantially affect individuals in resource-depleted areas. Considering that microsurgical practice is essential for resident training and serves as a recruitment tool to attract medical students to surgical subspecialties, increasing the accessibility of microsurgical training is necessary. In order to address the gap in microsurgery education, there has been a call to develop more accessible and available simulators [1]. When creating a microsurgical simulator, there are two main components to address in the design process: the equipment (i.e., replicating a surgical microscope) and the surgical specimen replication. Stereomicroscopes serve as effective replicates of surgical microscopes and can be incorporated in a microsurgical training laboratory at a cost of less than $500 [2]. In designing surgical specimens, the use of 3D printing [3] and human placenta [4] have been suggested and are designed to replicate microvascular anastomoses in neurosurgery. However, these simulators require more formalized infrastructure to be in place and are likely inaccessible for individuals in resource-depleted areas. On the other hand, the use of common day items, including eggs [5] and oranges [6], is easily accessible for trainees to obtain. Despite the cost-effectiveness of these materials, practicing microsurgery in a meaningful and effective manner remains limited without access to a surgical microscope. To address these limitations, Bedi MS et al. developed the Lazy Glass Microsurgical Trainer, which requires a smartphone, a set of reflective prism glasses, and a cardboard box for an overall cost of $5 minimum [7]. Though other resources have employed the use of smartphones for practicing microsurgical techniques [8], the Lazy Glass Microsurgical Trainer creates the element of “eye-hands blind” orientation (not being able to see your hands through the simulator), which provides a more representative experience of using a microscope for the trainee. Given the ability to create a more accessible means of microsurgical practice and the potential to increase medical student interest in microsurgery, we provide our experience conducting a randomized control trial with the Lazy Glass Microsurgical Trainer. In this study, we sought to determine the effectiveness of microsurgery simulation when utilizing the Lazy Glass Microsurgical Trainer.

## Materials and methods

Subject recruitment

Our experimental design and protocol were approved by the Institutional Review Board of the Mayo Clinic (IRB# 20-013230). Participants from all four years of medical school were recruited from the Mayo Clinic Alix School of Medicine and stratified randomized (matched by year) into control or treatment groups. At the time of recruitment, participants were made aware of the study requirements, including the required pre-testing session for all participants and the training sessions for the experimental group. Participants signed informed consent forms prior to study commencement.

Study design

**Figure 1:**
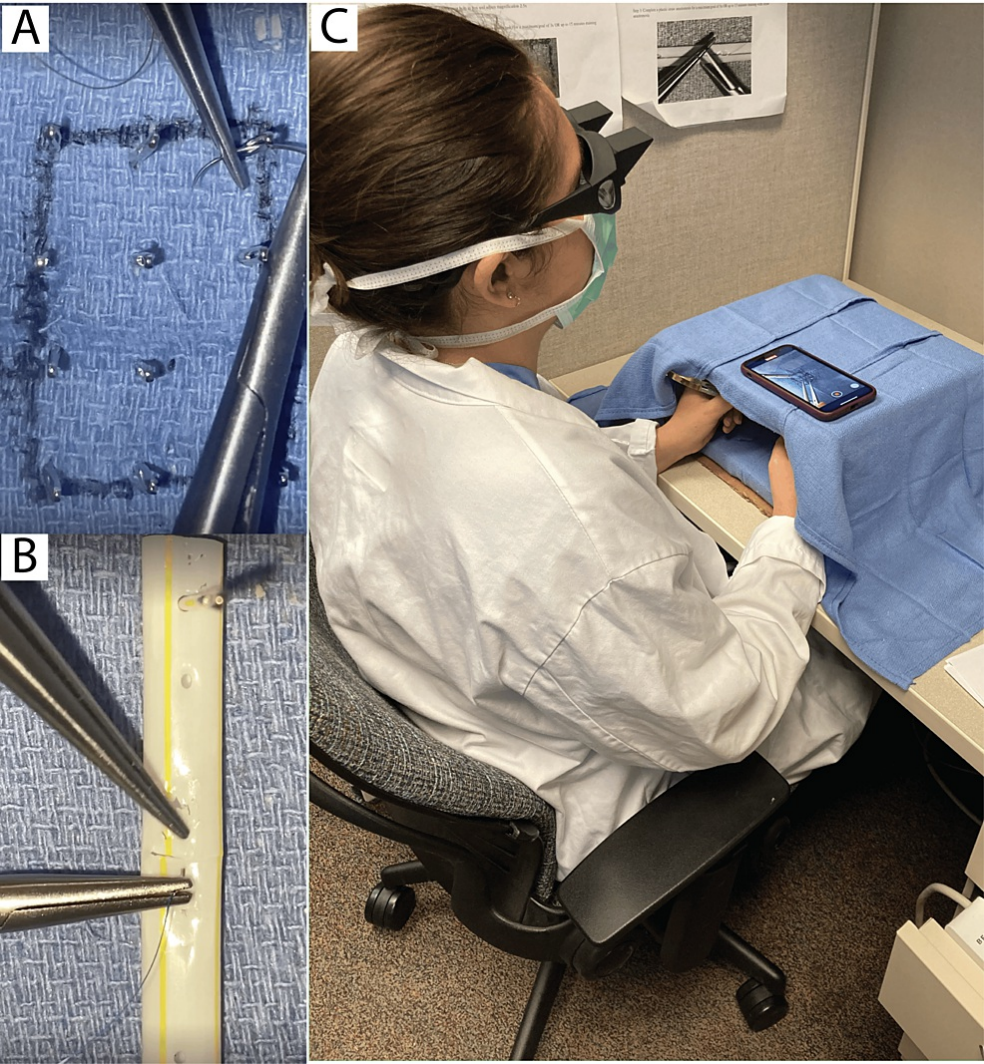
Tasks performed during training sessions. A. Maze task, where participants guide a 5-0 silk suture through a series of sewing needles in sequence. B. Straw anastomosis task, requiring participants to tie four knots circumferentially around a plastic straw. C. A photograph taken during a typical Lazy Box training session.

From 2021 to 2022, subjects were randomly allocated in blocks (based on medical school year) to control or treatment groups by a member of the study design team (Kimberly Wang). Each participant was assigned a number for the study team to remain blinded throughout the study duration, except for the designated group allocator. Every participant participated in the pre-and-post study tasks consisting of ligating two cut vessel loops (x2 sutures for each loop), a red rubber tube (x4 sutures for each), finished by microsurgical anastomosis on a synthetic blood vessel tube kit (x6 sutures for each). All this was performed under the operating microscope described previously [9]. Participants were timed under these conditions with the following time allocations for each task: 10 minutes (vessel loop), 15 minutes (red rubber tube), and 35 minutes (synthetic vessel). Participants completed a Swedish Occupational Fatigue Inventory (SOFI) survey and Surgery Task Load Index (SURG-TLX) to assess their fatigue during microscope use (each taken immediately after pre and post-tests). Each participant filled out a pre-test survey taken prior to the pre-test and a post-test survey taken after completion of the post-test to assess their prior level of surgical experience and experience using the LazyBox [7,9,10]. If assigned to the experimental cohort, subjects trained for three days per week over four weeks, choosing their own times, on the LazyBox [7,9,10] located within the medical school building (Figure 1). The training tasks included: 1) passing a 5-0 silk needle suture continuously through 12 adjacent needle eyes three times (Appendices 1 and 2) and completing a plastic straw anastomosis (Appendix 2). After four weeks of training (or not, depending on their cohort allocation), participants underwent a post-test, which involved the same tasks as the pre-test. They then completed the survey again. Participants were evaluated based on task completion (number of ties thrown), time to completion, and quality, which was assessed by testing the fluid's patency and leakage through the tubes [9]. These assessments were conducted by the study design team.

Statistical analysis

All statistics were performed in R version 4.1.0. Given the treatment and control group sizes of less than 25, Kruskal-Wallis tests were used to compare numeric outcomes. Statistical significance was set at a threshold of 0.05.

Participant characteristics

A total of 28 students enrolled in our trial. Out of these, 24 completed the trial, one dropped out prior to randomization, and three dropped out after randomization, resulting in an 86% (n=24) participation rate. The participants who completed the trial represented a variety of class years, including first-year students (n = 12), second-year students (n = 7), third-year students (n = 4), and one fourth-year student. Only three students in our cohort reported having no prior experience with general surgical skills (Table 1).

**Table 1:** Demographic characteristics of all students who completed the trial. MS: Medical School; MD: Doctor of Medicine; PhD: Doctor of Philosophy; OMFS: Oral and Maxillofacial Surgery.

Demographic	Control group (n=12)		Treatment arm (n=12)	
	n	%	n	%
Year				
MS1	6	50	6	50
MS2	4	33.3	3	25
MS3	2	16.7	2	16.7
MS4	0	0	1	8.3
Program				
MD only	7	58.3	9	75
MD-PhD	4	33.3	1	8.3
MD-Other Advanced Degree	0	0	1	8.3
OMFS	1	8.3	1	8.3
Surgical Skills Experiences Prior to Study				
None	1	8.3	2	16.7
<5	7	58.3	7	58.3
5-10	2	16.7	1	8.3
11-15	1	8.3	1	16.7
>15	1	8.3	1	8.3

## Results

Comparison of objective task performance metrics

**Figure 2:**
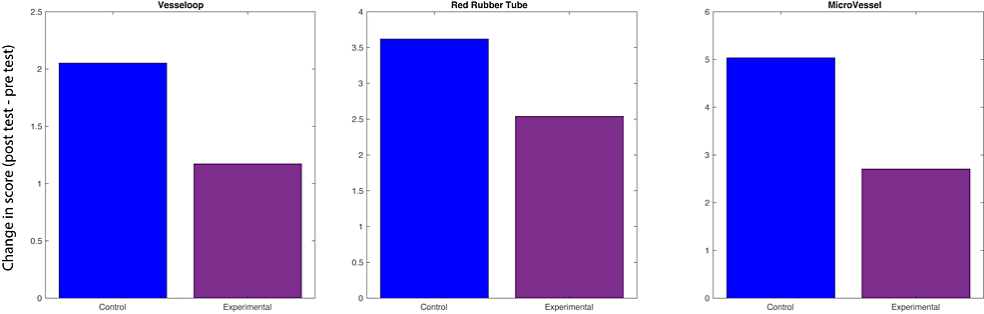
Task performance. Differences in performance between control and experimental groups for the vessel loop, red rubber tube, and microvessel tasks. The values represented by the bars are the differences between the mean pre-test and post-test scores of the control and experimental groups. No significant differences (p < 0.05) in performance were found between the groups for the vessel loop, red rubber tube, or microvessel tasks.

Participants in both the treatment and control groups were compared based on their performance in the vessel loop ligation, catheter macroanastomosis, and synthetic vessel microanastomosis tasks. In the vessel loop ligation task, the treatment arm participants showed a 1.2-point increase (SD = 2.6) in performance score between initial and repeat testing, compared to a 2.1-point increase (SD = 2.4) in the control arm (p = 0.39). For the macroanastomosis task, both groups demonstrated mean improvements, with a 3.4-point increase in the control group (SD = 4.1) and a 2.9-point increase in the treatment group (SD = 3.6) (p = 0.74). In the synthetic vessel microanastomosis task, both groups showed improvements, with a 5.4-point increase in the control group (SD = 8.3) versus a 2.9-point increase in the treatment group (SD = 5.6) (p = 0.30) (Figure 2).

There was no statistically significant difference in mean improvement in overall score between the control group (+10.9, SD = 10.8) and experimental group (+6.6, SD = 7.5) (Table 2).

**Table 2:** Performance comparison. Comparison of the change in performance scores between control and treatment groups across various tasks. No significant differences (p < 0.05) in performance were found between the groups. The data are presented as Mean (SD).

	Control (N=12)	Treatment (N=12)	Total (N=24)	P-value
Change in Vessel Loop Task Performance				0.453
Mean (SD)	2.140 (2.355)	1.342 (2.569)	1.741 (2.445)	
Range	-1.700-5.000	-3.467-4.796	-3.467-5.000	
Change in Red Rubber Task Performance				0.735
Mean (SD)	3.354 (4.130)	2.930 (3.551)	3.151 (3.783)	
Range	-2.620-9.570	-1.000-9.580	-2.620-9.580	
Missing	0	1	1	
Change in Synthetic Vessel Task Performance				0.299
Mean (SD)	5.417 (8.302)	2.919 (5.586)	4.168 (7.037)	
Range	-12.973 - 17.440	-5.657 - 13.490	-12.973 - 17.440	
Change in Overall Score				0.389
Mean (SD)	10.911 (10.753)	6.589 (7.471)	8.844 (9.384)	
Range	-5.053-26.610	-2.150-20.330	-5.053-26.610	
Missing	0	1	1	

Comparison of reported task fatigue

**Figure 3:**
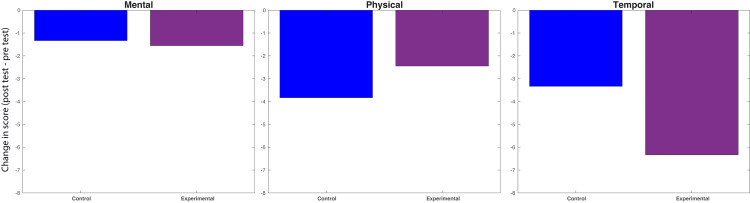
SURG-LTX index scores. Average change in SURG-TLX index scores between control and experimental groups. All questions in the SURG-TLX are categorized under mental, physical, and temporal challenges, with lower scores indicating reduced stress in these categories. The values represented by the bars show the difference between the mean pre-test and post-test scores of the control and experimental groups. No significant differences (p < 0.05) in performance were found between the groups for the mental, physical, and temporal indices. SURG-TLX: Surgery Task Load Index.

The SOFI and SURG-TLX surveys were utilized to assess task fatigue at initial and repeat testing. Participants in the treatment arm who completed SOFI surveys (n = 10) reported an average decrease of 5.0 points (SD = 7.6) in physical discomfort between initial and repeat testing, compared to a 2.3-point decrease (SD = 5.7) in the control arm (n = 12) (p = 0.82). Similarly, there was an average decrease of 4.8 points (SD = 7.4) in reported physical exertion in the treatment arm, in contrast to a 1.4-point decrease (SD = 2.5) in the control arm (p = 0.47). Additionally, the treatment arm experienced a 4.8 point decrease (SD = 6.1) in reported lack of energy, compared to a 5.1 point decrease in the control arm (p = 0.74) (Figure 3).

**Figure 4:**
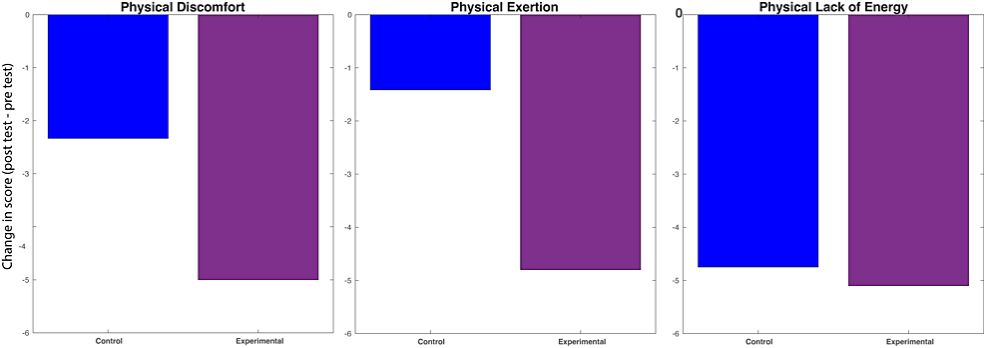
SOFI index scores. Average change in SOFI index scores between control and experimental groups. All questions in the SOFI are categorized under challenges related to physical discomfort, exertion, and lack of energy, with lower scores indicating less physical stress in these categories. The values represented by the bars show the differences between the mean pre-test and post-test scores of the control and experimental groups. No significant differences (p < 0.05) in performance were found between the groups for the physical discomfort, exertion, or lack of energy indices. SOFI: Swedish Occupational Fatigue Inventory.

Participants in the treatment arm who completed SURG-TLX surveys (n = 8) experienced an average decrease of 1.5 points (SD = 4.5) in the reported mental demands of completing the task between initial and repeat testing, compared to a 1.3-point decrease (SD = 6.5) in the control group (n = 12) (p = 0.82). Regarding the physical demands of the task, the treatment arm reported a 2.4-point decrease (SD = 5.3) between initial and repeat testing, in contrast to a 3.8-point decrease (SD = 3.9) in the control group (p = 0.48). Lastly, for the temporal demands of the task, the treatment group saw a 7.1-point decrease (SD = 7.4), as opposed to a 3.3-point decrease (SD = 5.2) in the control group (p = 0.23) (Figure 4).

## Discussion

In this randomized educational intervention trial, we objectively assessed the effectiveness of a training regimen with a low-fidelity microsurgery simulator. A total of 24 students completed our trial, including an assessment of microsurgical skills at baseline and after six weeks in a control group that received no training versus an experimental group that participated in a weekly training regimen on a low-fidelity microscopy simulator. While we observed mean improvement in both groups, there were no statistically significant differences in measured objective and subjective task performance metrics in the experimental and control groups. The results of our trial have several key implications for structuring microsurgical education curricula and designing educational intervention trials.

Previous microsurgery education trials

To date, microsurgery education trials can generally be categorized as bench trials, animal models, human models, live animal models, augmented reality models, and training curricula [11]. Bench models involve using inorganic material to train basic microsurgical techniques in a cost-effective manner (anastomoses, suturing, etc.). Our trial is based on inorganic materials and would thus qualify as a bench model. Amongst previous bench model trials, latex gloves and gauze have been used to train suturing [12-15]. Some groups used cardboard [15-17] to train basic knot-tying skills. Several papers have been published on the proper placement and guidance of microsuture through beads [18-20] and sewing needles [18]. Others have addressed the skill of microanastomosis using Japanese noodles, polyethylene tubes, or silicon tubes, most similar to our microvessel anastomosis model [21-23]. Most of the literature regarding novel microsurgical training techniques is descriptive in nature, and very few randomized controlled trials exist. Those that do exist have used training courses that consist of both inorganic materials (e.g., gauze, tubes, beads) and organic animal models (e.g., rat vessels). One randomized controlled trial of 32 participants ranging from medical students to post-graduate residents compared the order of consecutive biangulation and triangulation anastomoses of rat vessels after a five-day training course, which used a variety of inorganic and organic materials [24]. One randomized controlled trial with 39 medical student participants compared chicken femoral artery anastomosis quality after training with a tabletop microscope, a tablet camera, or a set of jeweler's microscopes for four weeks using sewing needles and vinyl gloves [14]. Another randomized controlled trial with 46 medical student participants compared microsurgical and a combination of macro/microsurgical training prior to being evaluated during a macro-surgical skills test and a microsurgical skills test. To our knowledge, no baseline test (pre-test) was performed, and skills were assessed only using a macrosurgical skill task for each group. A microsurgical task was conducted in the experimental group, but the scores were not compared to those of the control group as they did not perform the microsurgical task [25]. With 38 surgical residents (PGY1-3), this randomized controlled trial compared four surgical training sessions using a Penrose drain, a synthetic artery model, and turkey arteries either compressed in one day or spread across four weeks [14,26]. Although each trial addresses unique questions regarding microsurgical training, there is a dearth of randomized controlled trials utilizing training paradigms with purely inorganic material-based models.

Lessons learned and limitations with the LazyBox trial

There are several lessons to be learned from our trial and limitations to bear in mind while interpreting our results or designing future educational intervention trials. Firstly, we found considerable variation in the learning curves of students who participated in our trial, as evidenced by the large ranges in the change in task performance scores. Of note, some students in the experimental group even demonstrated decreased performance compared to their baseline. This may be due to a lack of translation of skills learned in the LazyBox to the operative microscope. A less satisfying explanation could be that some students experienced a negative shift in their post-test performance simply due to chance. This speaks to the need for greater data collection of task performance at multiple time points and for a longer duration. Another key point for consideration is the difference between deliberate and non-deliberate practices. It is possible that students within our training groups who completed the weekly regimen of self-practice with the low-fidelity simulator were not deliberate in their practice and, without a source of feedback, ended up repeating similar mistakes in their performance tests. This could be better teased apart by collecting data on the practice sessions themselves.

Most medical students enrolled were in their first and second years (M1 and M2), possessing minimal surgical experience, particularly in advanced microscopy. Consequently, enrolling more formally educated medical students or junior residents might provide a more suitable group for microscopy-simulator training. Additionally, there's a possibility that our tasks, designed to simulate nerve and vascular anastomosis, may not be effective training exercises for medical students or may not be highly reproducible. Moreover, our metrics and surveys might not be sufficiently sensitive to detect subtle improvements. Further training would be required before concluding that the LazyBox microscopy simulator has limited value. This project serves as another example, contrary to the satirical 'parachute trial' analogy, that not all training exercises may yield absolute benefits, underscoring the importance of high-quality randomized studies in educational settings where feasible.

## Conclusions

In this large-scale, randomized educational intervention trial involving medical students, we observed no statistically significant difference in microsurgical skill acquisition or improvement in comfort levels between students who participated in weekly training sessions using a low-fidelity microscopy simulator and those who did not. While low-fidelity simulators may be attractive to educators in resource-limited settings, it is crucial to rigorously evaluate these interventions to confirm their effectiveness in enhancing skill acquisition. This ensures that both trainee time and departmental resources are optimally utilized.

## Appendices

Appendix 1

Appendix 2
